# Mechanistic assessment and ablation of left ventricular assist device related ventricular tachycardia in patients with severe heart failure

**DOI:** 10.3389/fphys.2023.1086730

**Published:** 2023-04-12

**Authors:** Felix Hohendanner, Matthias Bock, Julian Keznickl-Pulst, Vesna Furundzija, Sebastian Scholz, Doreen Schöppenthau, Yuriy Hrytsyna, Volkmar Falk, Burkert Pieske, Gerhard Hindricks, Evgenij Potapov, Jin-Hong Gerds-Li

**Affiliations:** ^1^ Deutsches Herzzentrum der Charité, Klinik für Kardiologie, Angiologie und Intensivmedizin, Berlin, Germany; ^2^ Charité—Universitätsmedizin Berlin, Corporate Member of Freie Universität Berlin and Humboldt-Universität zu Berlin, Berlin, Germany; ^3^ German Center for Cardiovascular Research (DZHK), Partner Site Berlin, Berlin, Germany; ^4^ Department of Cardiology, German Heart Center Berlin, Berlin, Germany; ^5^ Deutsches Herzzentrum der Charité, Klinik für Herz, Thorax- und Gefäßchirurgie, Berlin, Germany; ^6^ Translational Cardiovascular Technologies, Institute of Translational Medicine, Department of Health Sciences and Technology, Swiss Federal Institute of Technology (ETH) Zurich, Berlin, Germany

**Keywords:** ventricular tachycardia, LVAD, RF ablation, heart failure, bench to bedside

## Abstract

**Aims:** Left-ventricular-assist-devices (lvad) are an established treatment for patients with severe heart failure with reduced ejection fraction (HF) and reduce mortality. However, HF patients have significant substrate for ventricular tachycardia (VT) and the lvad itself might be pro-arrhythmogenic. We investigated the mechanism of VT in lvad-patients in relation to the underlying etiology and provide *in silico* and *ex-vivo* data for ablation in these HF patients.

**Methods and Results:** We retrospectively analyzed invasive electrophysiological (EP) studies of 17 patients with VT and lvad. The mechanism of VT was determined using electroanatomical, entrainment and activation time mapping. Ischemic cardiomyopathy was present in 70% of patients. VT originated from the lvad region in >30%. 1/6 patients with VT originating from the lvad region had episodes before lvad implantation, while 7/11 patients with VT originating from other regions had episodes before implantation. Number and time of radiofrequency (RF)-ablation lesions were not different between VTs originating from the lvad or other regions. Long-term freedom from VT was 50% upon ablation in patients with VT originating from the lvad region and 64% if ablation was conducted in other regions. To potentially preemptively mitigate lvad related VT in patients undergoing lvad implantation, we obtained *in silico* derived data and performed *ex-vivo* experiments targeting ventricular myocardium. Of the tested settings, application of 25 W for 30 s was safe and associated with optimal lesion characteristics.

**Conclusion:** A significant percentage of patients with lvad undergoing VT ablation exhibit arrhythmia originating in close vicinity to the device and recurrence rates are high. Based on *in silico* and *ex-vivo* data, we propose individualized RF-ablation in selected patients at risk for/with lvad related VT.

## 1 Introduction

Terminal heart failure with reduced ejection fraction (HF) is associated with severe morbidity and mortality. Mechanical left ventricular assist devices (lvad) are often used to provide these patients with a last resort destination therapy and as bridge to transplant. Novel devices use centrifugal continuous flow and have been associated with improved outcomes in patients with heart failure and lvad for destination therapy. However, HF patients with lvad have significant myocardial substrate for ventricular arrhythmias and the apical inflow canula and the suture line of the fixation ring of the lvad itself might contribute to pro-arrhythmogenic substrate (Shi et al., 2022).

A considerable number of patients undergoing lvad implantation are in heart failure due to ischemic cardiomyopathy. These patients often show substantial *ad hoc* left ventricular scar that can be related to delayed onset focal or macro-reentry tachycardia during the course of their disease. However, lvad implantation itself creates left ventricular scar at the junction of cannula and left ventricle and a potential mechanical stimulus for the induction of pro-arrhythmic afterdepolarizations.

A high percentage of patients undergoes invasive endo- and/or epicardial ablation procedures to reduce VT burden. During these procedures different electrophysiology maneuvers allow determining the origin of the VT. RF ablation often not only targets the critical scar related isthmus, but also areas with substrate that might sustain arrhythmia in close vicinity to the lvad cannula. Procedures are long, challenging and often yield heterogeneous long term results due to the complexity of this set of patients.

Aim of the current study was to investigate the mechanism of ventricular arrhythmias in lvad patients and to facilitate characterization of this special patient cohort. For this purpose we quantitatively investigated VT in 17 lvad patients that underwent an electrophysiology study. We further assessed recurrence rates as well as clinical parameters to ultimately aid in refining a set of patients potentially benefiting from ablation strategies aiming at myocardium adjacent to the lvad cannula.

Based on *in silico* and *ex-vivo* data we also provide evidence for a potentially preventive epicardial ablation strategy during surgical lvad implantation or prospective epicardial ablation after lvad implantation in selected patients. For this purpose, we first developed an *in silico* model to define optimal target regions for point by point ablation in potential patients with lvad and VT. We then employed an *ex-vivo* pig model to obtain safe RF ablation settings for our proposed set of ablation lesions in patients with or at risk for lvad cannula related VT.

## 2 Materials and methods

### 2.1 Clinical human study

We retrospectively analyzed all patients with lvad (Medtronic HeartWare^TM^ VAD, Medtronic, Dublin, Ireland and Abbott HeartMate III^TM^, Abbott Laboratories, Chicago, United States) presenting for VT ablation with available high density 3D electroanatomic substrate maps between 03/2019 and 01/2022 at the German Heart Center Berlin (*n* = 17). VT ablation was performed on a case by case basis with the primary aim of internal defibrillator therapy mitigation or hemodynamic stabilization.

Procedures were performed either under sedation or in general anesthesia and all patients were receiving antiarrhythmic therapy as per electrophysiology/intensive care unit team before and after ablation. Heparin was administered during all procedures to achieve an acute clotting time of >300 s. Map and ablation catheters were advanced *via* femoral access sites either through venous and trans-septal or trans-aortic sheaths into the left ventricle. 3D electroanatomic high density mapping was performed using Carto-3 (Biosense Webster, California) and either a Deca-Nav or Pentaray catheter (Biosense Webster, California). The CARTOUNIVU module of Carto-3 (Cano et al., 2016) was used for X-ray guided left ventricular mapping and regions <1.5 mV were considered “low voltage”/scar. In patients presenting in sinus rhythm, VT was induced with programmed ventricular stimulation. Substrate, entrainment and pace mapping were used in most cases to facilitate detection of the critical isthmus. Moreover, whenever feasible, fractionated signals or late potentials were targeted with additional ablation lesions at the operators’ discretion. Ablation lesions were created with irrigated RF with a power of up to 40 W at 43°C. The procedure endpoint was termination and non-inducibility of the clinical VT. VT was defined as having an lvad origin (i.e., lvad associated) if the critical isthmus involved myocardium visibly adjacent to the lvad cannula (activation mapping) or entrainment mapping (isthmus exit in lvad vicinity) at times combined with pace-mapping. In cases classified as lvad associated VT, ablation was performed in close vicinity to the cannula.

Recurrence rates were determined during the 20 ± 3 months follow-up after ablation. Positive recurrence was defined as follows: VT documented with either 12-lead ECG, CIED documentation or a death certificate indicating sudden cardiac death. Clinical parameters (i.e., ischemic *versus* non-ischemic) as well as the occurrence of VT before lvad implantation were obtained from admission letters/medical reports available at time of presentation for the electrophysiology examination.

The study was approved by the local ethics committee (EA2/124/22).

### 2.2 In-silico simulation

Simulation: We demonstrated the effect of transmural lesion patterns *via in silico* simulation. The model was simulated as a time-discrete finite element system with a finite number of states. The state vectors for all mesh points (cells) were calculated at n equidistant time intervals $t_{0},t_{1},t_{2},\ldots t_{n}\in I$ with Δt=50 μs for the simulated time interval $I=\left[0;500\right]\;ms$ .

Geometrical model: A half ellipsoid was used as crude geometrical approximation of the human ventricle. The ellipsoid was stretched in three dimensions to match the average size of a human left ventricle with height h=86 mm and diameters $d_{x}=47\;mm$ and $d_{y}=48\;mm$ , respectively (Pravdin et al., 2014).

Lvad insertion is represented by apical sparing with a diameter of d=40 mm . A FEM mesh with approximately 15000 cells was generated. Each mesh point represents a “cluster of cardiomyocytes”, while the mesh edges connect neighboring clusters and the faces constitute a left-ventricular endocardial surface as a two-dimensional manifold with edges at apex and base.

**FIGURE 1:**
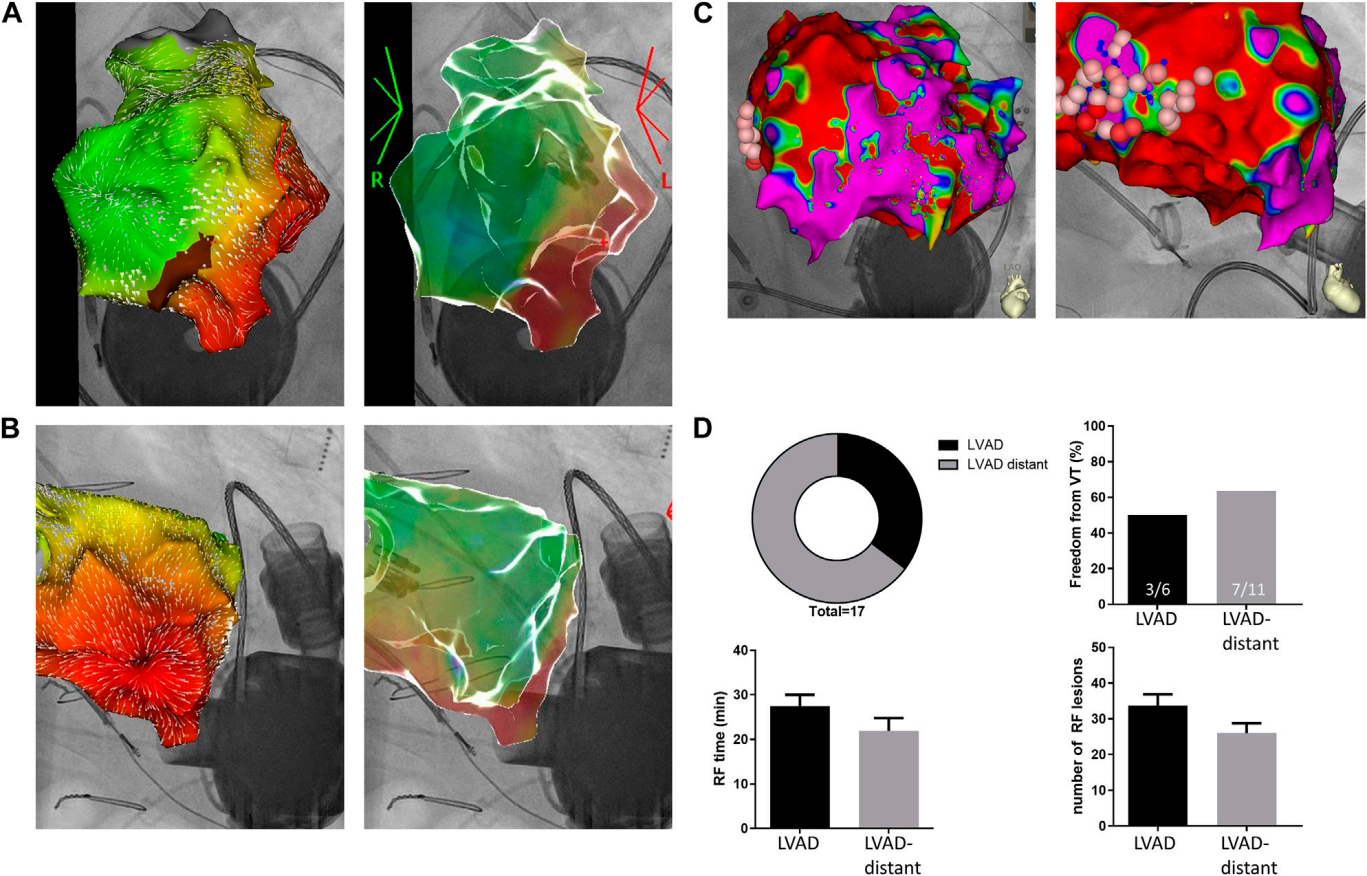
**(A and B)**. LVAD related VT: Anterior-posterior AP; **(A)** and left anterior oblique LAO; **(B)** view of the left ventricle. CARTOUNIVU overlay of 3D electroanatomic map [local activation time and carto-confidence (white arrows) map] and X-ray image. Mapping was performed during an ongoing focal VT from the LVAD insertion site. **(C)**. Non-LVAD related VT patient: AP (left) and LAO (right) view of the left ventricle. CARTOUNIVU overlay of 3D electroanatomic map (bipolar voltage map; red areas <1.5 mV) with marked ablation sites distant from the LVAD cannula. **(D)**. Distribution of LVAD associated and non-LVAD associated VT in LVAD patients and freedom from VT (top). Time of radiofrequency ablation (RF time) as well as number of radiofrequency ablation lesions (bottom). Patients had either an LVAD with LVAD-related or non-LVAD related VTs (LVAD-distant).

Excitation model: Cell cluster excitation was modeled as a finite state machine (FSM). Cluster states and state transitions are shown in Figure 1A. State transitions occur during pacing and after the respective states’ predefined durations elapse: Cells remain in resting state for an unlimited duration, in excitation/depolarization state for 20 ms , in absolute refractory period for 180 ms , in relative refractory period for 50 ms .

Conduction model: The propagation of cluster excitation was modeled by assigning a time delay to every mesh edge according to the respective length and the desired conduction velocity. Conduction velocity was set to 41cm/s - which is lower than in healthy patients but to be expected for patients with cardiomyopathy (Aronis et al., 2020). Every cluster in excited state excites all neighboring clusters, i.e., clusters directly connected *via* mesh edges, if the latter are in resting state when the respective mesh edge time delay has elapsed. Conduction velocity at points representing transmural RF ablation lesions was set to 0 at a lesion diameter of 7 mm.

Pacing: Pacing is implemented by selecting a time point for pacing as well as the cells to be paced. If selected cells are in resting state at the specified time, their state is set to excited.

Lvad induced arrythmia were modelled as premature excitation at the presumed contact surface of lvad and left ventricle. We simulated arrythmia by pacing the most apical cells every 300 ms to mimic VT at 200 bpm. Normal sinus rhythm was simulated by pacing the most basal cells every 800 ms ( 75bpm ) not taking into account altered electrical properties owing to the specific excitation conduction system (i.e., His-Purkinje network).

Software: OpenSCAD was used to generate the three-dimensional model of the left ventricle. Gmsh was used to yield simulation meshes suitable for FEM analysis. Simulation was developed using OpenMP and OpenCL. Rendering was carried out using VTK, PyVista and ParaView (software references available on request).

### 2.3 *Ex-vivo* simulation

For the *ex-vivo* experiments, explanted pig hearts were placed in a chamber containing physiologic saline solution at room temperature (*n* = 3 hearts). RF lesions were generated using a CELSIUS (Biosense Webster, California) 3.5 mm tip catheter with a power of 25, 35 or 45 W at 43°C (15 mL/min irrigation). The ablation catheter was manually directed to slabs of myocardium in a perpendicular fashion as previously described (Parwani et al., 2020). After RF delivery, lesion cross-sections were quantified (width, depth) and the adjacent myocardium was macroscopically inspected. “Steam pops” were defined as sudden local temperature rises leading to audible myocardial rupture at the ablation site.

### 2.4 Statistics

All data is presented as mean ± standard error mean and analysis was performed in a blinded-fashion. GraphPad Prism was used for statistical inference and plotting (GraphPad Software, San Diego, California United States). To test for differences, ANOVA analysis, Binomial or Boschloo’s tests were performed. A *p* < 0.05 indicates significant statistical difference.

## 3 Results

**FIGURE 2:**
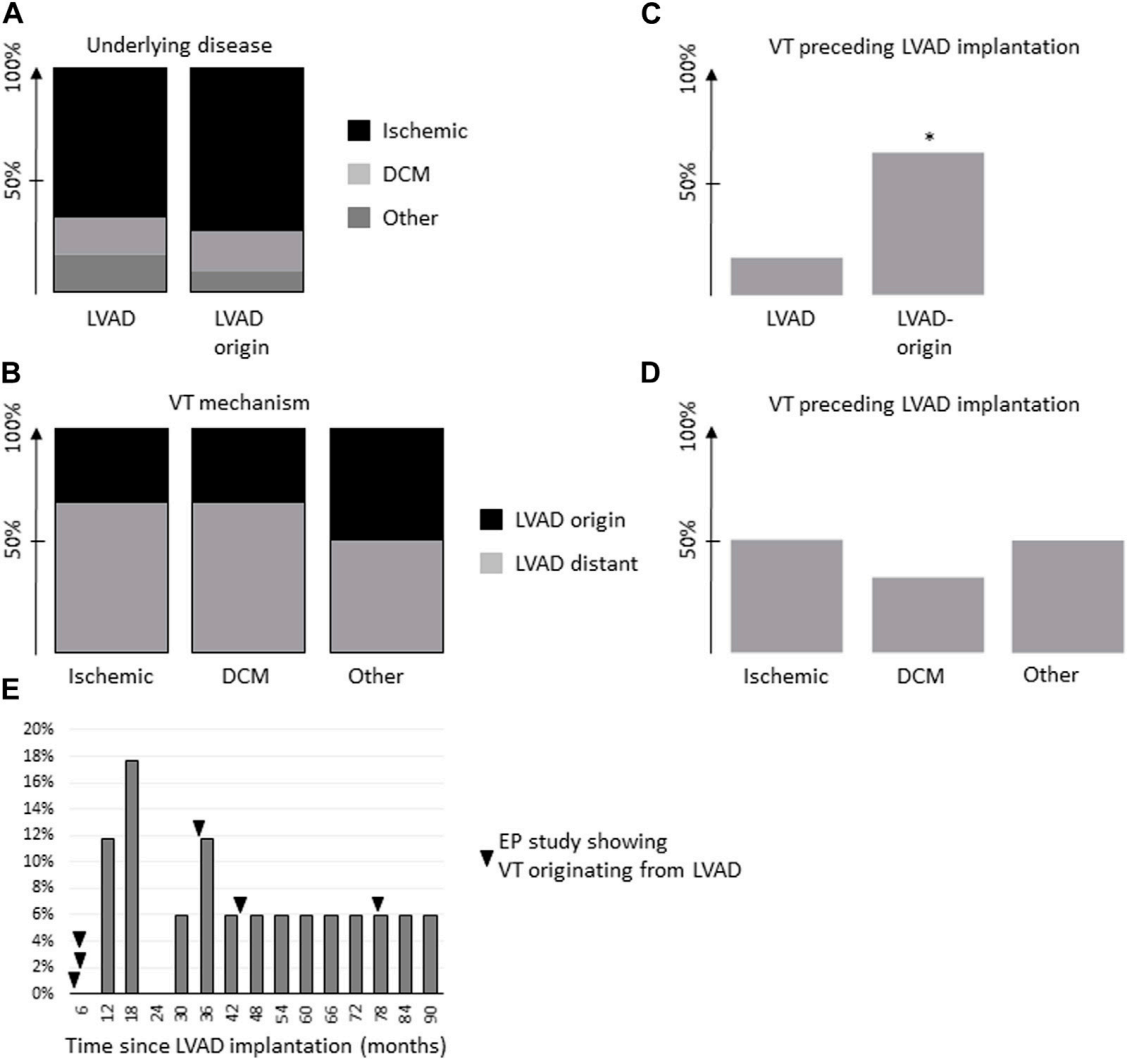
**(A)**. Underlying disease in patients with VT (VT) that was determined to be LVAD associated (“LVAD”) or not LVAD associated (“LVAD-distant”). **(B)**. VT mechanism, i.e., ischemic, non-ischemic dilatative cardiomyopathy (DCM) or other in relation to the respective VT - LVAD association. C and **(D)**. Percentage of patients with VT preceding LVAD implantation in relation to their respective VT–LVAD association **(C)** or underlying disease entity **(D)**. **(E)**. Histogram of time since LVAD implantation for all patients at end of follow-up. Time-points of EP cases with LVAD associated VT as indicated (black arrow). *p < 0.05 for non-LVAD-association of VT.

We investigated a clinical cohort of 17 lvad patients that underwent RF ablation for VT to determine the underlying VT mechanism and the respective lvad association. Baseline clinical parameters showed no significant difference between lvad patients with and without VT related to the device. Mean time since lvad implantation was 47 ± 13 and 40 ± 7 months in patients with VT originating from the lvad or other VT, respectively (Table 1). The main mechanism of arrhythmia determined during electrophysiological examination was not lvad associated in 11 out of 17 patients. In our study cohort, six out auf 17 cases of VT were directly lvad associated (i.e., focal, adjacent to the lvad insertion or macro-reentry, around the lvad cannula; Figure 1). VT induction in lvad associated cases was either achieved using programmed ventricular stimulation (S3 in two cases), spontaneous induction (*n* = 2) or ongoing at presentation (*n* = 2). VT was induced in not LVAD associated cases as follows: S2 or S3 in five cases, spontaneous induction in two cases and ongoing at presentation in one case. A mere substrate map indicated a critical isthmus in three not LVAD associated cases. All but two patients with VT originating from the cannula presented with ischemic cardiomyopathy. All but one patient with ischemic cardiomyopathy and VT from the vicinity of the lvad-cannula had received some form of coronary intervention or coronary bypass graft addressing the left anterior descending artery. One patient with ischemic cardiomyopathy and lvad associated arrhythmia had reported episodes of VT before lvad implantation (1/6 patients), whereas seven out of 11 patients with lvad distant VT had reported episodes even before lvad implantation. Upon further investigation of our study collective, most patients showed underlying ischemic cardiomyopathy in both lvad associated and not lvad associated groups (Figures 2A,B). However, patients without lvad associated VT were significantly more likely to show VT before lvad implantation (Figure 2C). Moreover, lvad association of VT significantly correlated with the individual pre lvad VT status, i.e., patients with VT before lvad implantation (*n* = 8) were significantly more likely to show VT not originating from the lvad (*n* = 7) during the invasive electrophysiology study. Vice-versa, patients without prior VT (*n* = 9) were 11% more likely to develop lvad associated rather than lvad distant VT and 83% of the patients with lvad associated ventricular arrhythmia had no prior documented VT.

**TABLE 1:** Patient characteristics. *p < 0.05 for non-LVAD-association of VT.

	VT: LVAD origin (n = 6)	VT: Other origin (n = 11)
Age (y)	56 ± 4	61 ± 2
Sex (male)	83% (5)	100% (11)
Time since implant (m)	47 ± 13	41 ± 7
Time between implant and EP study (m)	27 ± 12	21 ± 7
Follow-up after EP study (m)	20 ± 5	19 ± 4
Antiarrhythmics	100% (6)	100% (11)
Underlying disease ischemic	67% (4)	72% (8)
VT before LVAD implantation	17% (1)	64% (7)*
LVEF prior to LVAD	20 ± 4	15 ± 1
Catecholamine-support during EP study	0% (0)	18% (2)
Cycle length VT (ms)	408 ± 30	436 ± 40
Death	0%	18% (2)

Within the study cohort, freedom from VT was 50% upon ablation in patients with VT originating from the lvad region and 64% if ablation was conducted in non-lvad regions (Figure 1). Number of RF ablation lesions (34 ± 3 and 26 ± 3 lesions in lvad associated and not lvad associated, respectively) and RF time (27 ± 3 and 22 ± 3 in lvad-associated and not lvad associated, respectively) were not significantly different between the groups. Two patients with VT originating not from the lvad and none of the VT cases originating from the lvad died during follow-up.

Most patients with lvad associated VT presented with ischemic cardiomyopathy and substrate was identified in vicinity to the cannula in all patients.

**FIGURE 3:**
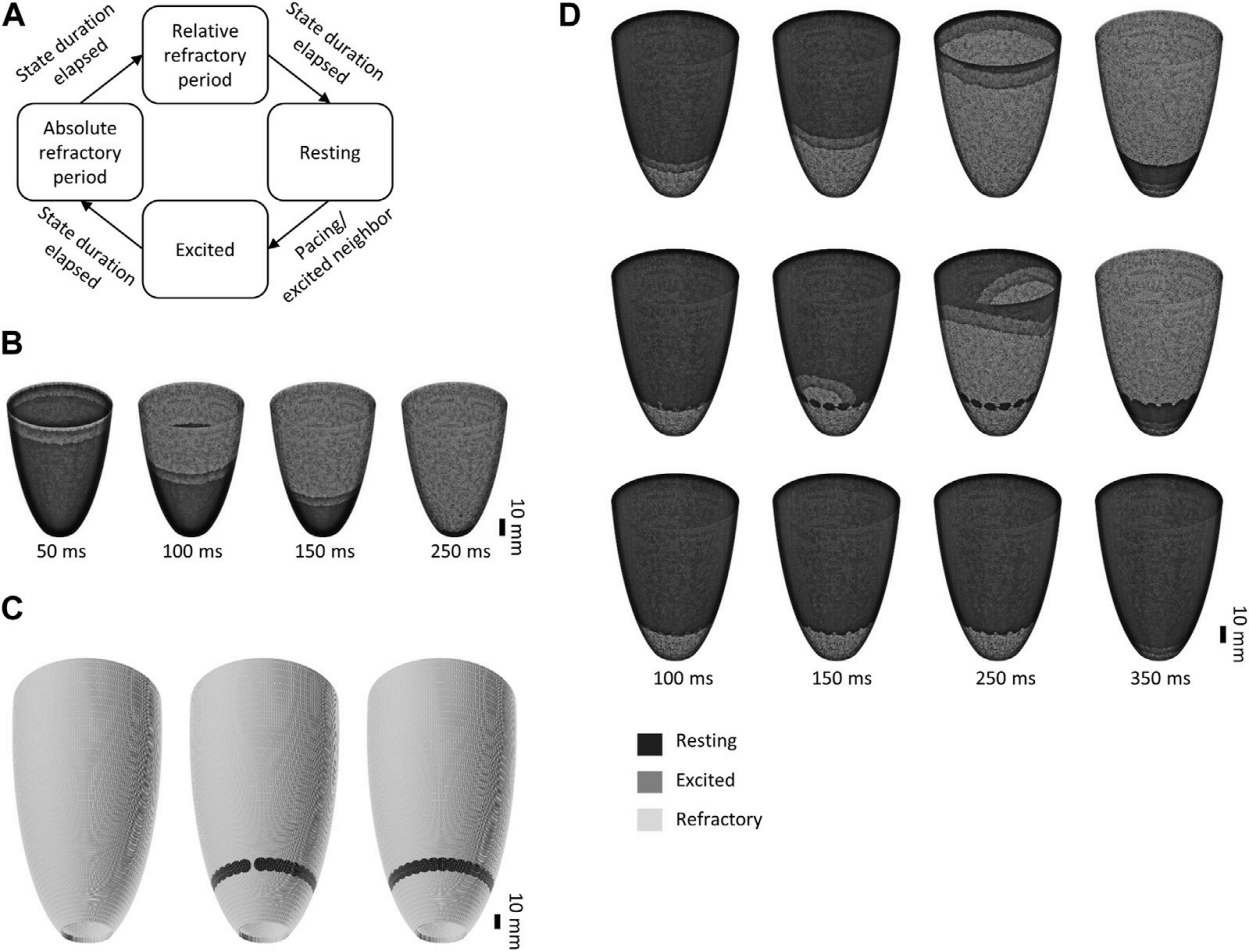
Simplified two-dimensional *in silico* model of the left ventricle. **(A)**. Cell cluster states and state transitions in the finite state machine used to generate the model. **(B)**. Electrical propagation during sinus rhythm (cycle length 800 ms). **(C)**. 3D mesh incorporating no (left), incomplete (center) and a complete, i.e., continuous set of ablation lesions. **(D)**. Simulation of VT (cycle length 300 ms) originating from the apical LVAD insertion site without (top), with incomplete (center) or with complete (bottom) sets of ablation lesions.

We therefore developed a simple two-dimensional *in silico* model of the left ventricle to determine the optimal ablation site in the clinical scenario of VT involving the apex, i.e., originating from a potential lvad cannulation site, of the left ventricle for preemptive surgical transmural RF ablation during lvad implantation or prospective epicardial ablation after lvad implantation. Electrical propagation from the basis towards the apex mimicking sinus rhythm without taking into account the specific cardiac conduction system is shown in Figure 3B to illustrate the models capabilities. Potential RF ablation lesions were introduced into the simulation with and without continuity/transmurality 20 mm above the envisioned apical lvad insertion. Lesion width was set to 7 mm, resembling the mean lesion width obtained *in-vivo* (see below). As shown in Figure 3D, the distinctive epicardial substrate modification with apical “encirclement” was able to mitigate electrical propagation towards the ventricular basis during VT *in silico*.

**FIGURE 4:**
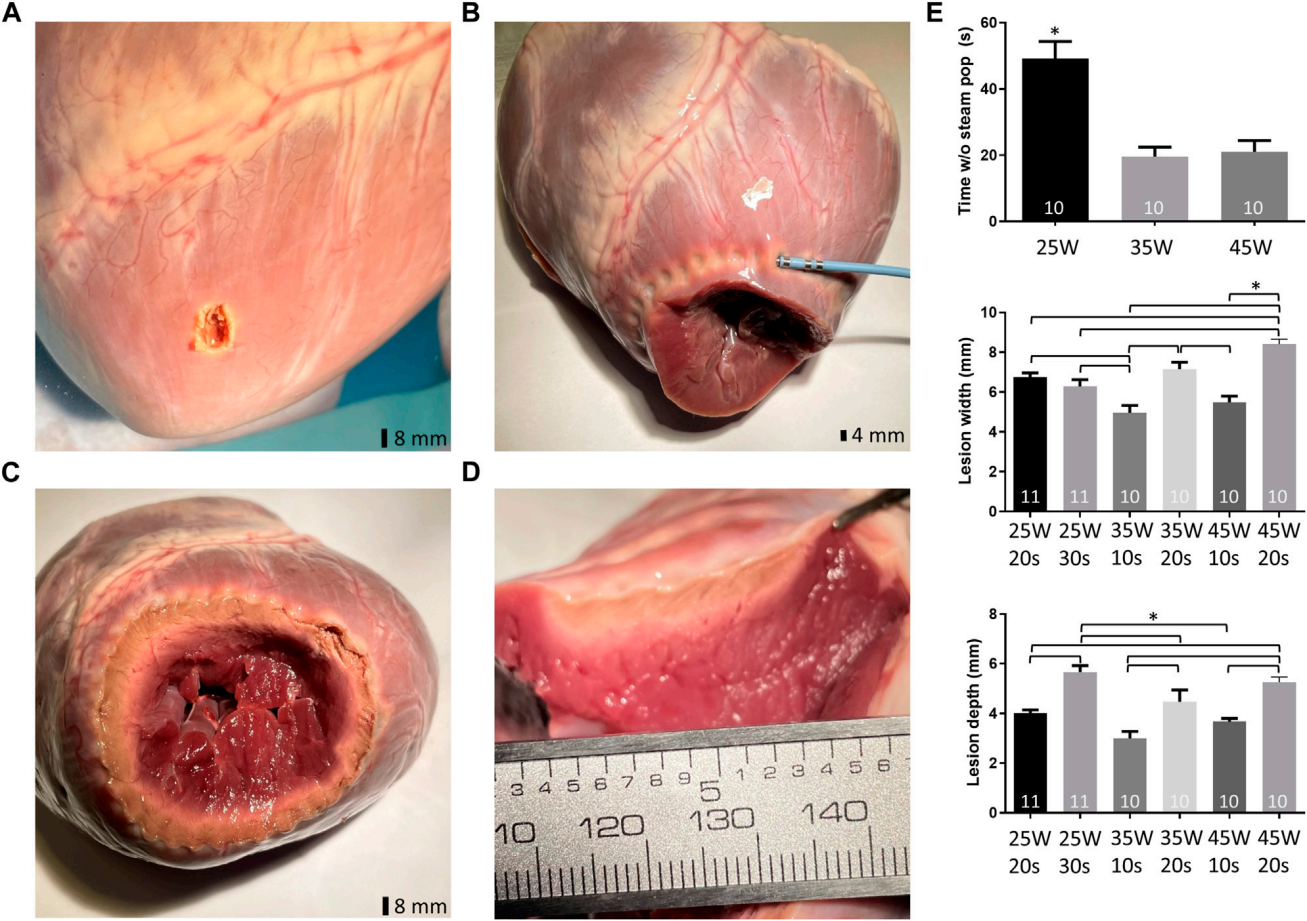
Lesion characteristics *ex-vivo* (pig). **(A)**. Example for “steam pop” related myocardial injury after epicardial ablation. **(B)**. Encirclement using RF-ablation (Celsius catheter^®^) of the prospective LVAD implantation site/cannulation site. **(C)**. Cross-section of the left ventricle with continuous set of RF lesions. **(D)**. Continuity of RF-lesions (manual inter-lesion distance 5 mm). **(E)**. Time without “steam pop” i.e., safe ablation time with 25, 35 and 45 W using the Celsius 3.5 mm tip catheter at 43°C and 15 mL/min irrigation (top left). Lesion depth and widths using the RF settings as indicated (center, bottom).

Last, we performed *ex-vivo* experiments (Figure 4) in pig hearts to determine optimal epicardial RF ablation settings to potentially target myocardium adjacent to the lvad cannula for preemptive surgical transmural radiofrequency ablation during lvad implantation or prospective epicardial ablation after lvad implantation. Using the CELSIUS catheter, RF application with 25 W for 20 s yielded a mean lesion width of 6.8 ± 0.2 mm. Lesion width was unchanged when energy was delivered for 30 s (6.3 ± 0.3 mm). However, lesion depth significantly increased upon application of 25 W for 30 s to 5.7 ± 0.3 mm (from 4.0 ± 0.1 mm at 25 W for 20 s). Lesion width and depth significantly increased further with 35 and 45 W for 10 or 20 s Respectively. Epicardial ablation was safe (i.e., no steam-pop) for up to 49 ± 5 s with 25 W, for up to 20 ± 3 s with 35 W and for up to 21 ± 3 s with 45 W. However, the earliest steam pops occurred after 14, 10 and 9 s with 25, 35 and 45 W, respectively.

## 4 Discussion

The study investigated VT mechanisms in lvad patients in relation to the underlying etiology and preexisting VT and provides *in silico* and *ex vivo* data to determine potential ablation parameters in selected patients with lvad and VT or lvad and at risk for VT.

In the present study, the lvad cannula was determined to be a relevant contributor to the observed VT in more than 30% of all lvad cases that underwent ablation. Previous work found the LVAD cannula as VT “culprit” in 9% (Sacher et al., 2015) and 14% (Cantillon et al., 2012) of cases, respectively. Others reported an incidence of post-lvad implantation VT anywhere between 18% and 43% in cohorts of up to 100 patients as well as a 4.5 higher likelihood for VT onset upon lvad implantation, while a recent meta-analysis with a total of 110 patients reported cannula-related VT in 19.3% (Ziv et al., 2005; Anderson et al., 2019).

The variable results in different studies might potentially be owing to different time-points and heterogenous patient populations. In addition, most patients in the present study were in ischemic cardiomyopathy and the likelihood of a scar related critical VT isthmus in close vicinity to the lvad insertion is high. In our particular study cohort, the majority of patients with ischemic cardiomyopathy had received some form of coronary intervention or coronary bypass graft surgery addressing perfusion-demand imbalances in the left anterior descending artery prior to lvad implantation. This underscores that apical scars in the territory of the coronary arteries are present in a high percentage of patients irrespective of assist device related scar formation or other potentially assist device related VT culprits (Yokokawa et al., 2009). The sampling density of substrate mapping and its correlation to anatomical structures can be poor (Zhong et al., 2007)—an additional factor that might lead to an overestimation of VT cases associated with lvad, even though traditional entrainment mapping usually helps identifying the critical isthmus in clinical practice.

On the other hand however, in the present study, most patients with lvad associated arrhythmias had no reported VT episodes before lvad implantation. In line with this observation, patients with arrhythmias originating from other regions than the lvad where significantly more likely to have reported VT episodes even before lvad implantation and baseline VT rates in patients with end-stage HF are high (Connolly et al., 2000).

The apical inflow cannula and the suture line of the fixation ring of the lvad might contribute to pro-arrhythmogenic substrate and a mechanistic VT involvement in some patients is likely. However, ventricular arrhythmias in lvad patients are potentially also mechanically induced through contact between the inflow cannula and the left ventricular wall. Others have attributed mechanical irritation to a mismatch between the lvad inflow and outflow leading to left ventricular unloading and direct contact of the between cannula and myocardium, an issue that can be readily fixed by echocardiography guided fluid resuscitation and reduction of lvad flow (Vollkron et al., 2007; Cantillon et al., 2012). This positional VT should however be observable rather early or at least at a fixed time-point (Ahmed et al., 2019) after lvad implantation. As shown in Figure 2E, lvad associated VT leading to the invasive EP study occurred throughout the after-lvad implantation period. Only one EP study was performed within 4 weeks after lvad implantation making positional VT a potential contributor. Yet the exact impact of positional VT remains elusive due to the regularly concomitant presence of pro-arrhythmogenic electrical substrate in our patient cohort.

The present work also provides *in silico* and *ex-vivo* data to determine optimal parameters for (preventive) epicardial ablation during or after surgical lvad implantation in selected patients. To visualize the *in silico* effect of circular transmural RF-ablation on the propagation of VT from the ventricular apex to base, we used a simplified model of cardiac electrophysiology and excitation propagation. Similar models have been employed successfully in the past and allow simulation without extensive computational necessities (Pagani and Manzoni, 2021). More complex simulation models incorporate representations of the ventricular tissue structure and simulate a multitude of electrophysiological parameters or mechanical actuation (Kummer et al., 2022; O'Hara et al., 2011) potentially allowing to even analyze ablation effects on a cellular level. However, qualitative visualization of the mere electric propagation allows studying the effect of transmural, continuous sets of RF lesions on triggered activity (but not macro-reentry). At the same time, a finite state machine allows to set the conduction velocity to a desired value, a necessity considering the regularly observed slowing of conduction in patients with preexisting left ventricular disease. A similar approach utilizing a finite state machine to model voltage dependent Ca^2+^ dynamics was, e.g., used by Sathar et al. (Sathar et al., 2014). The present approach provides evidence for the importance of transmural, continuous lesions to mitigate focal and reentrant (albeit only those types of reentry involving the lvad cannula) VT propagation towards areas without VT generating substrate.

In addition, we obtained RF ablation parameters to deliver first data for a potential future transfer of our observational and *in silico* results into individual patient care: We have previously established an *ex-vivo* model to study lesion characteristics in pig myocardium (Parwani et al., 2020). We used this model to generate epicardial RF lesions with an irrigated ablation catheter readily available in the surgical operation theatre. Lesion characteristics using 25 W for 30 s showed a mean width and depth of 6.3 ± 0.3 and 5.7 ± 0.3 mm, resp. MRI studies have determined the mean ventricular myocardial thickness at the mid-cavity level at 5.3 ± 0.9 mm and 6.3 ± 1.1 mm for women and men, respectively (Kawel et al., 2012). Others found slightly higher values potentially owing to the progressive thickening from apex to base. However, mean myocardial thickness with ischemic cardiomyopathy tends to decrease further and show increased heterogeneity (Katikireddy and Acharya, 2019). The proposed RF settings of 25 W for 30 s led to a lesion depth *ex-vivo* of 5.7 ± 0.3 mm. This would allow to create transmural lesions in an epicardial approach during a combined invasive electrophysiology/surgical procedure for lvad cannula insertion.

Using these ablation settings no-steam pops occurred for up to 49.2 ± 5.1 s. Our earliest steam pop occurred after 14, 10 and 9 s with 25, 35 and 45 W, respectively. Of note, these potentially safety relevant incidences were regularly associated with small (*ex-vivo* i.e., non-perfused) coronary vessels in close vicinity to the ablation site. For preemptive ablation in selected lvad patients potential side-effects of this treatment have to be considered: Local non-transmurality might have unwanted pro-arrhythmic effects and impaired blood supply upon ablation might negatively affect tissue healing adjacent to the lvad cannula implantation side. It would therefore be paramount to generate transmural lesions and a future study of the long-term effects of this intervention is warranted.

In summary, a significant percentage of patients with lvad exhibit VT originating from the lvad region and overall recurrence rates are high. VT propagation towards the basis of the heart is mitigated *in silico* upon distinctive substrate modification. Based on the presented *ex-vivo* data we propose epicardial RF-ablation with 25 W for 30 s per ablation point with a cooled catheter in selected patients. Using these parameters epicardial RF ablation during or after lvad implantation might be an option for selected patients at risk for or with lvad associated VT.

## Data Availability

The raw data supporting the conclusion of this article will be made available by the authors, without undue reservation.

## References

[B1] Ahmed A. Amin M. Boilson B. A. Killu A. M. Madhavan M. (2019). Ventricular arrhythmias in patients with left ventricular assist device (LVAD). Curr. Treat. Options Cardiovasc Med. 21 (11), 75. 10.1007/s11936-019-0783-7 31773322

[B2] Anderson R. D. Lee G. Virk S. Bennett R. G. Hayward C. S. Muthiah K. (2019). Catheter ablation of ventricular tachycardia in patients with a ventricular assist device: A systematic review of procedural characteristics and outcomes. JACC Clin. Electrophysiol. 5 (1), 39–51. 10.1016/j.jacep.2018.08.009 30678785

[B3] Aronis K. N. Ali R. L. Prakosa A. Ashikaga H. Berger R. D. Hakim J. B. (2020). Accurate conduction velocity maps and their association with scar distribution on magnetic resonance imaging in patients with postinfarction ventricular tachycardias. Circ. Arrhythm. Electrophysiol. 13 (4), e007792. 10.1161/CIRCEP.119.007792 32191131 PMC7196439

[B4] Cano O. Andres A. Osca J. Alonso P. Sancho-Tello M. J. Olague J. (2016). Safety and feasibility of a minimally fluoroscopic approach for ventricular tachycardia ablation in patients with structural heart disease: Influence of the ventricular tachycardia substrate. Circ. Arrhythm. Electrophysiol. 9 (2), e003706. 10.1161/CIRCEP.115.003706 26850881

[B5] Cantillon D. J. Bianco C. Wazni O. M. Kanj M. Smedira N. G. Wilkoff B. L. (2012). Electrophysiologic characteristics and catheter ablation of ventricular tachyarrhythmias among patients with heart failure on ventricular assist device support. Heart rhythm. 9 (6), 859–864. 10.1016/j.hrthm.2012.01.018 22293139

[B6] Connolly S. J. Hallstrom A. P. Cappato R. Schron E. B. Kuck K. H. Zipes D. P. (2000). Meta-analysis of the implantable cardioverter defibrillator secondary prevention trials. AVID, CASH and CIDS studies. Antiarrhythmics vs implantable defibrillator study. Cardiac arrest study hamburg. Canadian implantable defibrillator study. Eur. Heart J. 21 (24), 2071–2078. 10.1053/euhj.2000.2476 11102258

[B7] Katikireddy C. K. Acharya T. (2019). Myocardial segmental thickness variability on echocardiography is a highly sensitive and specific marker to distinguish ischemic and non-ischemic dilated cardiomyopathy in new onset heart failure. Int. J. Cardiovasc Imaging 35 (5), 791–798. 10.1007/s10554-018-01515-3 30594979 PMC6486529

[B8] Kawel N. Turkbey E. B. Carr J. J. Eng J. Gomes A. S. Hundley W. G. (2012). Normal left ventricular myocardial thickness for middle-aged and older subjects with steady-state free precession cardiac magnetic resonance: The multi-ethnic study of atherosclerosis. Circ. Cardiovasc Imaging 5 (4), 500–508. 10.1161/CIRCIMAGING.112.973560 22705587 PMC3412148

[B9] Kummer T. Rossi S. Vandenberghe S. Demertzis S. Jenny P. (2022). Embedded computational heart model for external ventricular assist device investigations. Cardiovasc Eng. Technol. 13, 764–782. 10.1007/s13239-022-00610-w 35292915 PMC9616791

[B10] O'Hara T. Virag L. Varro A. Rudy Y. (2011). Simulation of the undiseased human cardiac ventricular action potential: Model formulation and experimental validation. PLoS Comput. Biol. 7 (5), e1002061. 10.1371/journal.pcbi.1002061 21637795 PMC3102752

[B11] Pagani S. Manzoni A. (2021). Enabling forward uncertainty quantification and sensitivity analysis in cardiac electrophysiology by reduced order modeling and machine learning. Int. J. Numer. Method Biomed. Eng. 37 (6), e3450. 10.1002/cnm.3450 33599106 PMC8244126

[B12] Parwani A. S. Hohendanner F. Bode D. Kuhlmann S. Blaschke F. Lacour P. (2020). The force stability of tissue contact and lesion size index during radiofrequency ablation: An *ex-vivo* study. Pacing Clin. Electrophysiol. 43 (3), 327–331. 10.1111/pace.13891 32091133

[B13] Pravdin S. F. Dierckx H. Katsnelson L. B. Solovyova O. Markhasin V. S. Panfilov A. V. (2014). Electrical wave propagation in an anisotropic model of the left ventricle based on analytical description of cardiac architecture. PLoS One 9 (5), e93617. 10.1371/journal.pone.0093617 24817308 PMC4015904

[B14] Sacher F. Reichlin T. Zado E. S. Field M. E. Viles-Gonzalez J. F. Peichl P. (2015). Characteristics of ventricular tachycardia ablation in patients with continuous flow left ventricular assist devices. Circ. Arrhythm. Electrophysiol. 8 (3), 592–597. 10.1161/CIRCEP.114.002394 25870335

[B15] Sathar S. Trew M. L. Du P. O'Grady G. Cheng L. K. (2014). A biophysically based finite-state machine model for analyzing gastric experimental entrainment and pacing recordings. Ann. Biomed. Eng. 42 (4), 858–870. 10.1007/s10439-013-0949-5 24276722 PMC3972386

[B16] Shi J. Yu X. Liu Z. (2022). A review of new-onset ventricular arrhythmia after left ventricular assist device implantation. Cardiology 147 (3), 315–327. 10.1159/000524779 35483328 PMC9393833

[B17] Vollkron M. Voitl P. Ta J. Wieselthaler G. Schima H. (2007). Suction events during left ventricular support and ventricular arrhythmias. J. Heart Lung Transpl. 26 (8), 819–825. 10.1016/j.healun.2007.05.011 17692786

[B18] Yokokawa M. Tada H. Koyama K. Ino T. Hiramatsu S. Kaseno K. (2009). The characteristics and distribution of the scar tissue predict ventricular tachycardia in patients with advanced heart failure. Pacing Clin. Electrophysiol. 32 (3), 314–322. 10.1111/j.1540-8159.2008.02238.x 19272060

[B19] Zhong H. Lacomis J. M. Schwartzman D. (2007). On the accuracy of CartoMerge for guiding posterior left atrial ablation in man. Heart rhythm. 4 (5), 595–602. 10.1016/j.hrthm.2007.01.033 17467627

[B20] Ziv O. Dizon J. Thosani A. Naka Y. Magnano A. R. Garan H. (2005). Effects of left ventricular assist device therapy on ventricular arrhythmias. J. Am. Coll. Cardiol. 45 (9), 1428–1434. 10.1016/j.jacc.2005.01.035 15862414

